# HDCA suppresses exosomal lncRNA MSTRG171708 release to inhibit Treg-mediated immunometabolism in hepatocellular carcinoma

**DOI:** 10.1186/s43556-026-00550-0

**Published:** 2026-09-11

**Authors:** Feng Yang, Wenqiong Huang, Chongkai Fang, Chong Zhong, Chaoyuan Huang, Meijun Liu, Zongzhen Meng, Xiaoli Chen, Claudio Mauro, Aiping Lyu, Kenneth C. P. Cheung

**Affiliations:** 1 Department of Cardiology, Jiangxi hospital, The First Affiliated Hospital, Zhejiang University School of Medicine, Nanchang, Jiangxi Province China; 2 Phenome Research Center, Hong Kong Baptist University, Hong Kong, China; 3 Science and Technology Innovation Center, Guangzhou University of Chinese Medicine, Guangzhou, China; 4 Department of Biliary-Pancreatic Surgery, The First Affiliated Hospital of Guangzhou University of Chinese Medicine, Guangzhou, China; 5 Department of Gastroenterology, Guangdong Provincial Hospital of Chinese Medicine, Guangzhou, China; 6 Department of Inflammation and Ageing, College of Medicine and Health, Queen Elizabeth Hospital, University of Birmingham, Mindelsohn Way, Birmingham, B15 2WB UK

**Keywords:** Hyodeoxycholic acid, Extracellular vesicles, LncRNA MSTRG171708, Regulatory T cells, Immunometabolic reprogramming, HCC metastasis

## Abstract

Hepatocellular carcinoma (HCC) exhibits poor patient outcomes due to its propensity for metastasis, yet the mechanisms linking metabolic dysregulation and immune evasion remain unclear. This study reveals that reduced hyodeoxycholic acid (HDCA) levels, coupled with elevated tumor-derived extracellular vesicles (EVs) carrying the long non-coding RNA MSTRG171708, correlate with aggressive HCC progression and enhanced lung metastasis. Mechanistically, MSTRG171708-enriched EVs are internalized by regulatory T (Treg) cells, stabilizing the HIF-1α/PKM2/PHD3 axis and triggering a metabolic shift toward glycolysis, thereby augmenting Treg migratory capacity and fostering an immunosuppressive microenvironment conducive to metastasis. Furthermore, HDCA suppresses EVs release by transcriptionally downregulating RAB27 and SNAP23, key mediators of exosome biogenesis, and effectively curtails metastatic burden in vivo. Clinically, high MSTRG171708 levels in HCC patients are associated with decreased HDCA levels and increased Treg infiltration. These findings establish HDCA-mediated regulation of EVs trafficking as a critical link between epigenetic modulation and immunometabolic reprogramming, proposing HDCA restoration as a novel therapeutic strategy to impede HCC metastasis.

**Supplementary Information:**

The online version contains supplementary material available at https://doi.org/10.1186/s43556-026-00550-0.

## Introduction

Hepatocellular carcinoma (HCC), a leading cause of cancer-related mortality, predominantly arises in individuals with chronic liver diseases such as hepatitis virus-induced cirrhosis or nonalcoholic fatty liver disease (NAFLD) [1–3]. Despite therapeutic advances, HCC prognosis remains dismal due to its aggressive metastatic potential, driven by immune evasion and immunosuppressive tumor microenvironment (TME) remodeling [4]. Notably, regulatory T cells (Tregs) are found in increased abundance within HCC tissues, where they suppress cytotoxic anti-tumor responses, facilitate immune escape, and foster metastatic niche formation [5].

Bile acids (BAs) are metabolic signaling molecules synthesized in the liver that, beyond their classical roles in lipid digestion, are increasingly recognized as regulators of hepatic inflammation, immunity, and carcinogenesis [6–8]. Through activation of nuclear and membrane receptors such as farnesoid X receptor (FXR) and Takeda G protein-coupled receptor 5 (TGR5), BAs act as signaling molecules that shape hepatic homeostasis and anti-tumor immune responses [9, 10]. Recent studies highlight that dysregulated BA signaling contributes to HCC progression by modulating both tumor and immune cell function [11]. For instance, secondary BAs like deoxycholic acid (DCA) generally promote tumorigenic inflammation and immune evasion [12]. whereas others, such as ursodeoxycholic acid (UDCA) demonstrate anti-inflammatory and immune-regulatory properties [13]. Additionally, BAs have been found to regulate exosome biogenesis and extracellular vesicle (EV) cargo loading, processes vital to intercellular communication and immune regulation within the tumor microenvironment (TME) [14, 15]. Intriguingly, hyodeoxycholic acid (HDCA), a secondary BA and FXR receptor ligand, has been shown to ameliorate metabolic disorders such as atherosclerosis, diabetes, and NAFLD [6, 16], while its role in HCC progression and immune regulation remains unclear.

Long non-coding RNAs (lncRNAs) have emerged as key regulators of gene expression in cancer, influencing processes such as metabolism, proliferation, and immune responses [17]. Additionally, by being loaded into EVs, lncRNAs help reshape the TME via stromal-immune interactions [18]. LncRNAs such as Lnc-Tim3 promotes T cell exhaustion, leading to reduced cytotoxicity and impaired antitumor immunity [19]. EV-packaged lincRNA HIFAL derived from tumour-associated macrophages stabilizes HIF-1α protein by blocking its interaction with PHD2, thereby enhancing aerobic glycolysis in the TME [20]. However, the mechanisms linking EV-transported lncRNAs to immunosuppressive TME remodeling, particularly in Tregs, remain poorly defined.

Here, we hypothesize that HCC-derived EVs carrying lncRNA MSTRG171708 modulate Treg glycolytic metabolism and migration, thereby promoting immunosuppression and lung metastasis. Using orthotopic HCC models, we found that HDCA administration markedly alleviates hepatic pathological injury and suppresses primary tumor growth as well as distant lung metastasis. Mechanistically, the protective anti-HCC and anti-metastatic effects of HDCA rely on FXR suppression. HDCA inhibits EV biogenesis in an FXR-dependent manner by disrupting multivesicular body (MVB) fusion with the plasma membrane and blocks EV release from tumor cells. We further demonstrate that lncRNA MSTRG171708 in EVs competitively binds PHD3, blocking HIF-1α degradation and driving Treg glycolysis, migration, and premetastatic niche formation [21–23]. Clinically, reduced HDCA levels correlate with elevated MSTRG171708, increased Treg infiltration, and poor HCC outcomes. By uncovering HDCA’s role in regulating EV-lncRNA-Treg crosstalk, this study identifies FXR antagonism and HIF-1α/PKM2/PHD3 axis inhibition as novel strategies to block HCC metastasis.

## Results

### HDCA improves liver function and alleviates HCC pathological features

**Fig. 1:**
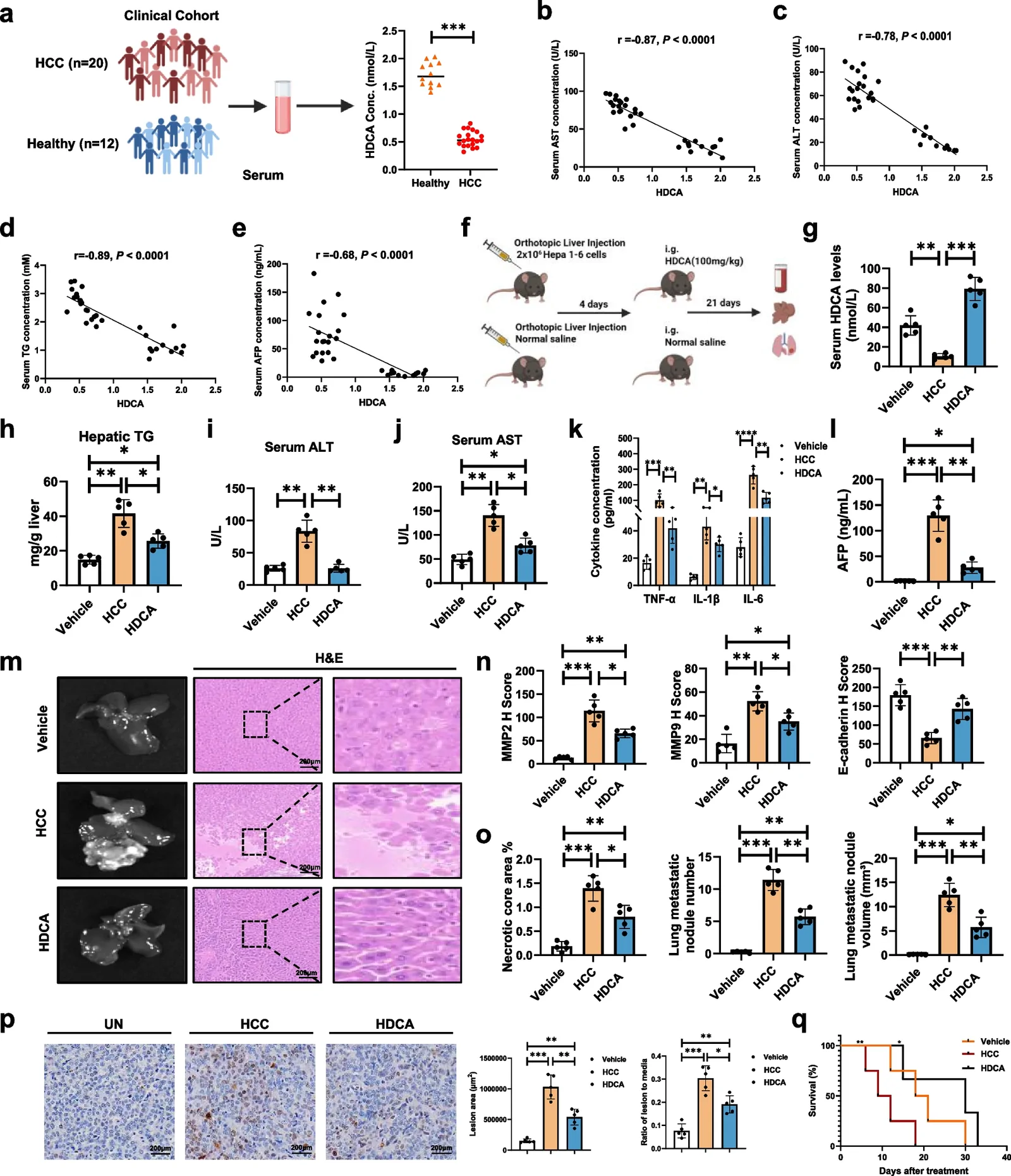
HDCA is reduced in HCC and HDCA treatment suppresses tumour progression in an orthotopic HCC mouse model.A schematic of clinical cohort and quantification of serum HDCA levels in healthy controls (*n* = 12) and HCC patients (*n* = 20). **b–e** correlation analyses between serum HDCA concentrations and clinical biochemical indices: (**b**) serum AST, (**c**) serum ALT, (**d**) serum TG, and (**e**) serum AFP. Negative correlations are observed in all parameters. F experimental design for orthotopic HCC mouse model and HDCA treatment. Mice received liver injections of Hepa1-6 cells and were treated with HDCA (100 mg/kg) or saline. G quantification of serum HDCA levels in mice after treatment with vehicle, HCC induction, or HDCA administration. **h–j** liver triglyceride content (**h**), serum ALT (**i**), and serum AST (**j**) in mice from different treatment groups. **k** cytokine concentrations (TNF-α, IL-1β, IL-6) in serum from various groups, indicating reduced inflammation upon HDCA treatment. **l** serum AFP levels in mice. **m** representative macroscopic and H&E-stained images of liver tissues from vehicle, HCC, and HDCA-treated groups, showing reduced tumor burden and improved histology with HDCA. **n** quantitative scores for MMP2, MMP9, and E-cadherin expression in liver tissues, with HDCA treatment restoring epithelial markers and reducing matrix metalloproteinases. **O** necrotic core area and lung metastatic nodule counts and volume in mice, indicating HDCA significantly decreases tumor necrosis and metastasis. **p **representative Ki67 immunohistochemical staining of liver sections, showing reduced lesion area and ratio of lesion to medial area after HDCA treatment. **q** Kaplan–Meier survival curves for mice in different treatment groups, showing improved survival following HDCA administration. Data are presented as mean ± s.d.. Comparisons between two groups were performed using unpaired two-tailed Student’s t-test. Comparisons among multiple groups were performed using one-way ANOVA with Tukey’s post hoc test. Correlation analyses used Pearson’s correlation coefficient. Survival differences were assessed using the log-rank test. *p < 0.05, **p < 0.01, ***p < 0.001. *n* = 5 independent biological replicates per group, unless otherwise specified

To elucidate BA alterations associated with HCC, we performed targeted profiling in sera from 20 HCC patients and 12 healthy controls, revealing a marked reduction of HDCA concentrations in HCC patients (Fig. 1a; Fig. S1a,b). Random forest analysis identified HDCA as the most discriminative BA between groups (Fig. S1c), indicating its potential as an HCC-associated biomarker. Notably, HDCA levels negatively correlated with serum AST, ALT, triglycerides, and AFP (Fig. 1b-e), suggesting that decreased HDCA is closely linked to heightened liver injury and tumour burden. Heatmap visualization further confirmed that HDCA exhibited the strongest and most consistent inverse associations with multiple clinical parameters among detected BAs (Fig. S1d).

To further validate these clinical findings, we employed an orthotopic HCC mouse model established by intrahepatic injection of Hepa1-6 cells, followed by administration of exogenous HDCA (100 mg/kg) or saline (Fig. 1f). Tumour induction resulted in a significant decrease in serum and hepatic HDCA concentrations, which were restored following HDCA supplementation (Fig. 1g; Fig. S1e). Additionally, HDCA treatment significantly reduced hepatic triglyceride content and serum ALT/AST activities (Fig. 1h-j), coinciding with decreased intrahepatic pro-inflammatory cytokines and tumor marker AFP (Fig. 1k,l). Macroscopically and histologically, HDCA administration resulted in reduced tumour size, improved hepatic morphology, and attenuated tumour invasion and metastasis, as evidenced by lower MMP2 and MMP9 scores and enhanced E-cadherin expression (Fig. 1m,n). Quantitative analyses revealed reductions in necrotic core area and pulmonary metastatic foci in HDCA-treated mice (Fig. 1o), while immunohistochemistry confirmed improved hepatic architecture and diminished lesion area (Fig. 1p). Consistent with these findings, in vitro molecular assays demonstrated that HDCA suppressed tumour cell proliferation and angiogenesis, and promoted apoptosis (Fig. S1f–i). Kaplan–Meier analysis further demonstrated that HDCA administration significantly prolonged survival of HCC-bearing mice (Fig. 1q). Collectively, these data demonstrate that HDCA is consistently diminished in HCC patients and experimental models, and are closely related to clinical indices of liver injury and tumor progression.

### HDCA enhances tumor suppression and anti-metastatic effects by inhibiting FXR

**Fig. 2:**
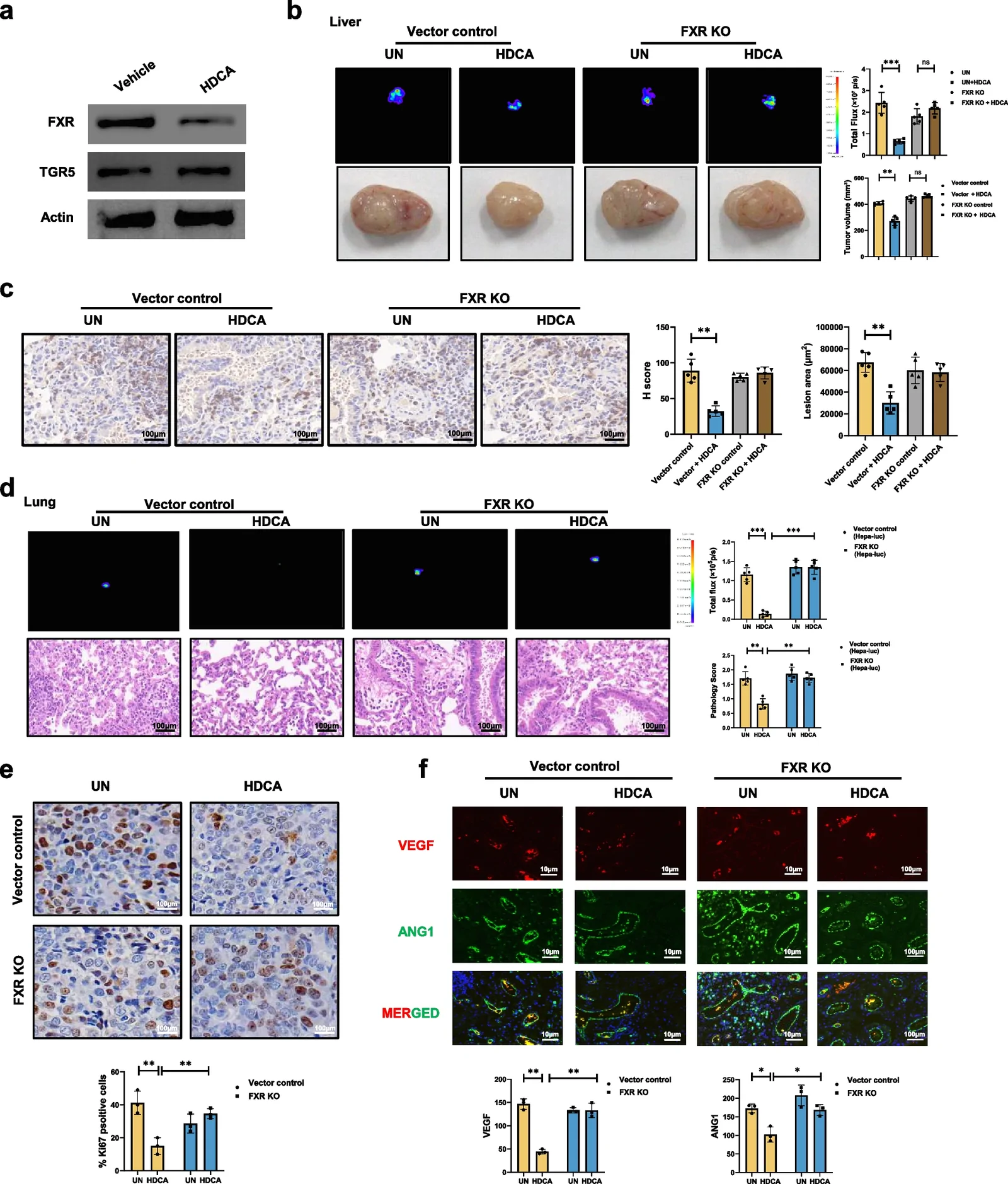
HDCA inhibits tumor growth, metastasis, and angiogenesis in an FXR-dependent manner.** a** Western blot analysis of FXR and TGR5 protein expression in liver tissues from mice treated with HDCA or vehicle control. **b** Representative bioluminescence images depicting hepatic tumor burden in vector control and FXR KO mice subjected to vehicle or HDCA treatment, with corresponding macroscopic views of excised liver tumors and tumor volume quantification shown below. **c** IHC staining of lung metastases in vector control and FXR KO mice following HDCA treatment, with quantification of H-score and lesion area.** d** Representative bioluminescence images to access lung metastases in vector control and FXR KO mice, with or without HDCA treatment. Corresponding H&E-stained lung sections and quantification of pathological scores are shown below. **e** Representative IHC images for Ki67 in liver tissues from vector control and FXR KO mice treated with or without HDCA, with quantification of Ki67-positive cells to evaluate proliferation. **f** Immunofluorescence staining of liver tissues for VEGF (red) and ANG1 (green) in vector control and FXR KO mice under different treatment conditions. Merged images show colocalization. Quantification of ANG1 and VEGF expression indicates reduced angiogenesis in vector control mice but not in FXR KO mice. Data are presented as mean ± s.d.. *n* = 3 for (a, e, f); *n* = 5 for (b-d). Statistical significance was determined by Student’s t-test or one-way ANOVA. *p < 0.05, **p < 0.01, ***p < 0.001

To elucidate the mechanistic basis of HDCA’s anti-tumor and anti-metastatic effects, we examined its impact on the expression of the BA receptors FXR and TGR5, both of which are central to BA-mediated signaling [24]. Quantitative analysis of liver tissues revealed that HDCA treatment significantly suppressed FXR levels, while TGR5 expression remained largely unchanged (Fig. 2a; Fig. S2a), implicating FXR as the primary mediator of HDCA’s action. Accordingly, HDCA reduced the mRNA expression of the FXR target genes SHP and BSEP, but not of DIO2, a TGR5-regulated gene (Fig. S2b-d), further supporting the specificity of HDCA’s effects on FXR signaling.

To directly test the role of FXR in HDCA-mediated tumor suppression, we employed both FXR-knockout (FXR KO) and FXR-overexpression (FXR OE) mouse models. In WT mice, HDCA administration led to a substantial reduction in hepatic tumor volume and bioluminescent tumor flux, accompanied by improvements in pathological features (Fig. 2b; Fig. S2e). In pulmonary metastasis models, HDCA dramatically decreased H scores, lesion area, metastatic nodule counts, Arginase-1 positivity, total luminescent signal, and comprehensive pathology scores, indicating potent inhibition of metastatic dissemination (Fig. 2c,d; Fig. S2f,g). Immunohistochemical and immunofluorescence analyses revealed that HDCA reduced the proportion of proliferating Ki67 + cells and suppressed angiogenesis markers ANG1 and VEGF in the tumour tissues (Fig. 2e,f). Strikingly, these anti-tumor and anti-metastatic effects were abolished in FXR KO mice, wherein HDCA failed to reduce tumor burden, metastatic spread, or associated pathological indices, demonstrating a requisite role for FXR inhibition in mediating HDCA efficacy.

Conversely, FXR OE exacerbated tumor progression, leading to significantly increased liver tumor volumes, tumor weight, H-scores, lesion areas, and metastatic bioluminescent flux compared to control mice (Fig. S2h–j). Interestingly, HDCA administration still attenuated primary and metastatic tumor burden in both vector control and FXR OE mice, although the effects were diminished in the FXR OE context, underscoring the FXR-dependent nature of HDCA’s anti-tumor actions and highlighting the contribution of FXR upregulation to tumor aggressiveness. Collectively, these findings demonstrate that HDCA suppresses tumor growth and metastasis primarily through targeted inhibition of FXR signaling.

### FXR modulates lncRNA expression and EV biogenesis in tumor cells

To investigate how FXR influences the TME in HCC, we examined long non-coding RNAs (lncRNAs) and EV production in tumor cells. Considering that lncRNAs are frequently selectively packaged into EVs (Fig. S4a) and play emerging roles in cancer progression and immune regulation [25], we profiled lncRNAs within plasma EVs from healthy control (HC) mice, metastatic HCC (HCC-LM) mice, and HCC-LM mice treated with HDCA (HCC-LM + HDCA), and confirmed EV purity prior to analysis (Fig. S4h). RNA-seq identified 5,077 novel lncRNAs and 23,478 mRNAs (Fig. S3a,b); among them, MSTRG171708 stood out as a top candidate, showing strong enrichment (> fivefold) in HCC EVs compared to controls and robust association with vesicle-related structures (Fig. S3c–l). RNA FISH localized MSTRG171708 predominantly to the cytoplasm in HCC cells, suggesting a post-transcriptional regulatory function (Fig. S3m).

**Fig. 3:**
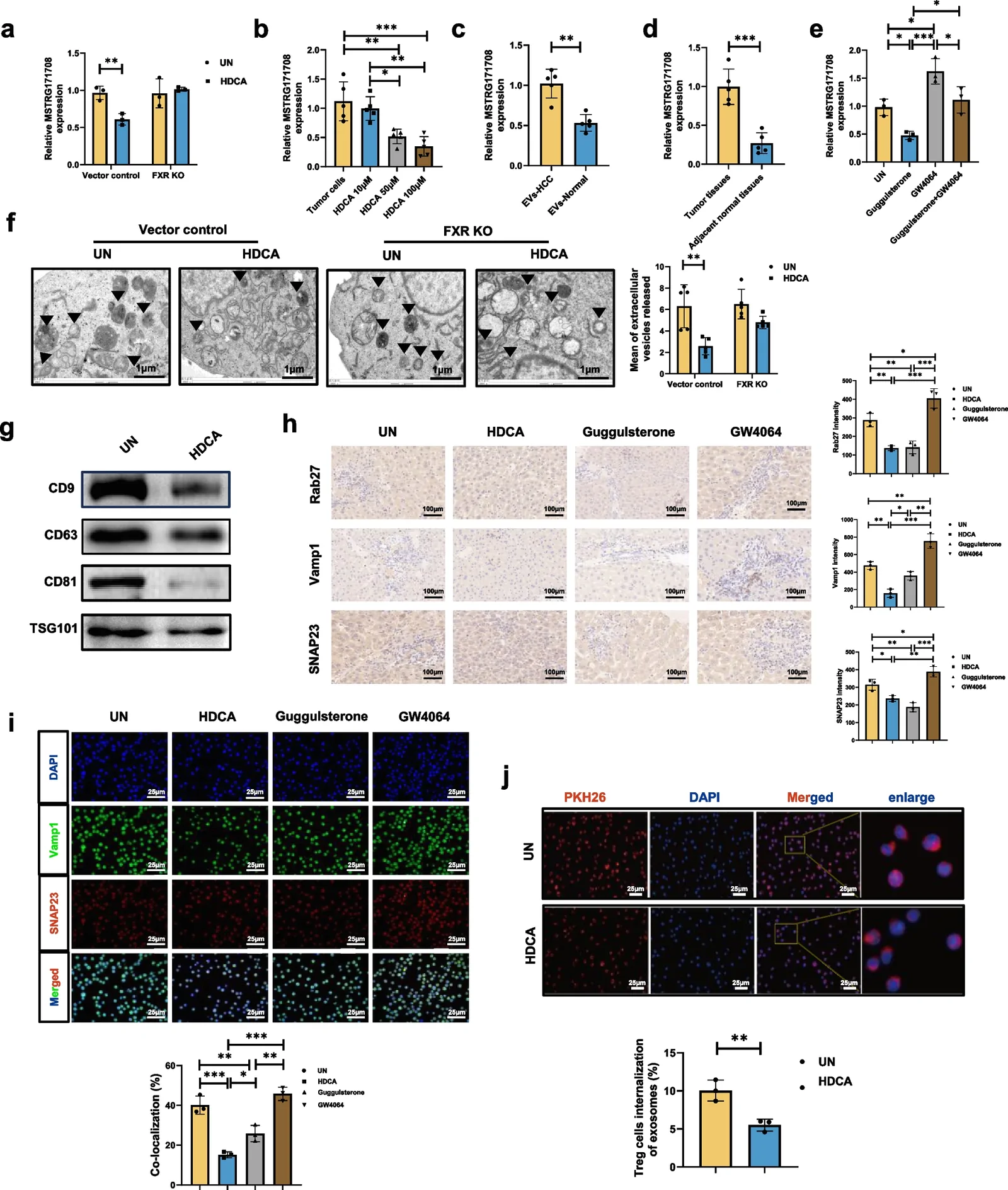
FXR regulates MSTRG171708 expression and extracellular vesicle (EV) biogenesis in tumor cells. **a** qRT-PCR analysis of MSTRG171708 levels in tumor cells under untreated and HDCA conditions, with or without FXR KO; HDCA downregulates MSTRG171708 expression in vector control.** b** Relative expression of lncRNA MSTRG171708 in tumor-derived EVs isolated from untreated or HDCA-treated (10 μM, 50 μM, and 100 μM) tumor cells, showing a dose-dependent reduction following HDCA treatment.** c** Quantification of lncRNA MSTRG171708 in serum-derived EVs from HCC-bearing and normal mice, confirming elevated expression of lncRNA in HCC-derived EVs relative to normal controls.** d** Analysis of lncRNA MSTRG171708 levels in EVs derived from HCC tumor tissues and adjacent normal tissues, revealing significantly increased expression in tumor tissue-derived EVs. **e** Quantification of lncRNA MSTRG171708 in tumor cell EVs following treatment with Guggulsterone (FXR antagonist) or GW4064 (FXR agonist) treatment, highlighting FXR-specific modulation of lncRNA incorporation.** f** Transmission electron microscopy (TEM) images of EVs isolated from tumor cells under indicated conditions; quantification of EV released shows a significant decrease in the number of EVs following HDCA treatment in the vector control group.** g** Western blot analysis of canonical EV markers (CD9, CD63, CD81, and TSG101) in EVs purified from untreated and HDCA-treated tumor cells; quantification reveals altered expression following HDCA treatment.** h** Immunohistochemical analysis of vesicular transport proteins (SNAP23, Vamp1, Rab27) in tumor tissues. Quantification shows that HDCA treatment leads to a decrease in their expression levels.** i** Immunofluorescence staining of EV marker proteins Vamp1 (green) and SNAP23 (red) in tumor cells under different treatments. Bar chart shows the quantification of Vamp1 and SNAP23 co-localisation. **j** Fluorescence microscopy showing PKH26-labelled EV uptake by Treg cells from untreated or HDCA-treated tumor cells; quantification of Treg cell EV internalization rates reveals that HDCA-treated EVs are less efficiently taken up. Data are presented as mean ± s.d..*n* = 3 for (a, e, g-j); *n* = 5 for (b-d, f). Statistical analysis was performed using two-tailed unpaired t-tests or one-way ANOVA with Tukey’s post hoc correction, as appropriate. Statistical significance is indicated as *p < 0.05, **p < 0.01, ***p < 0.001

Consequently, we investigated the regulation of lncRNA MSTRG171708 by FXR signaling in HCC. qRT-PCR results showed that HDCA stimulation led to a significant reduction in MSTRG171708 expression in vector control, whereas FXR KO abrogated this effect (Fig. 3a), implicating FXR as a key mediator in lncRNA transcriptional repression. Dose–response analysis revealed a graded decrease in MSTRG171708 expression in EVs isolated from HCC cells treated with increasing concentrations of HDCA (Fig. 3b). Remarkably, MSTRG171708 was selectively enriched in EVs from tumor-bearing mice compared to those from normal controls in both plasma (Fig. 3c) and tissue-derived EVs (Fig. 3d), highlighting disease-specific enrichment of this lncRNA in EV cargo. Pharmacological activation of FXR with GW4064 significantly increased MSTRG171708 expression, while this effect was counteracted by the FXR antagonist guggulsterone (Fig. 3e), reaffirming FXR-dependent regulation. Additionally, HDCA exposure suppressed EV secretion from tumor cells, as evidenced by transmission electron microscopy and decreased expression of canonical EV markers (Fig. 3f, g; S4b). TGR5 functional assays demonstrated that this regulator effect was primarily dependent on FXR rather than TGR5 (Fig. S4c, d). Selective depletion of CD81-positive vesicles and RNase protection assays further verified that MSTRG171708 is specifically encapsulated within EVs (Fig. S4e–g).

Further mechanistic insights revealed that FXR inhibition by HDCA or guggulsterone significantly diminished the abundance of Rab27, SNAP23, and Vamp1, key mediators of vesicular trafficking and membrane fusion. Restoration of their expression upon FXR activation by GW4064 implicated FXR as a regulator of these essential components (Fig. 3h). To investigate the functional consequences of this regulation, we assessed the co-localization of SNAP23 and Vamp1, finding that FXR blockade led to pronounced disruption of their spatial proximity, indicative of compromised SNARE complex assembly. This impairment was reversed by FXR agonism and was not observed in FXR-deficient cells, emphasizing the requirement for FXR in this process (Fig. 3i; S4i,j). Functional perturbation of vesicle release, achieved by knockdown of SNAP23 and Rab27, led to a marked decrease in lncRNA MSTRG171708, underscoring the central role of EV biogenesis in regulating lncRNA export (Fig. S4k,l). Moreover, HDCA treatment reduced the uptake of PKH26-labeled EVs by Treg cells, accompanied by decreased the levels of MSTRG171708, indicating compromised intercellular communication under FXR inhibition (Fig. 3j; S4m). Together, these data identify a previously unrecognized FXR–lncRNA–EV axis that governs intercellular communication and immune modulation in HCC.

### Synergistic inhibition of HCC progression and induce Treg exhaustion by targeted MSTRG171708 silencing and HDCA treatment

**Fig. 4:**
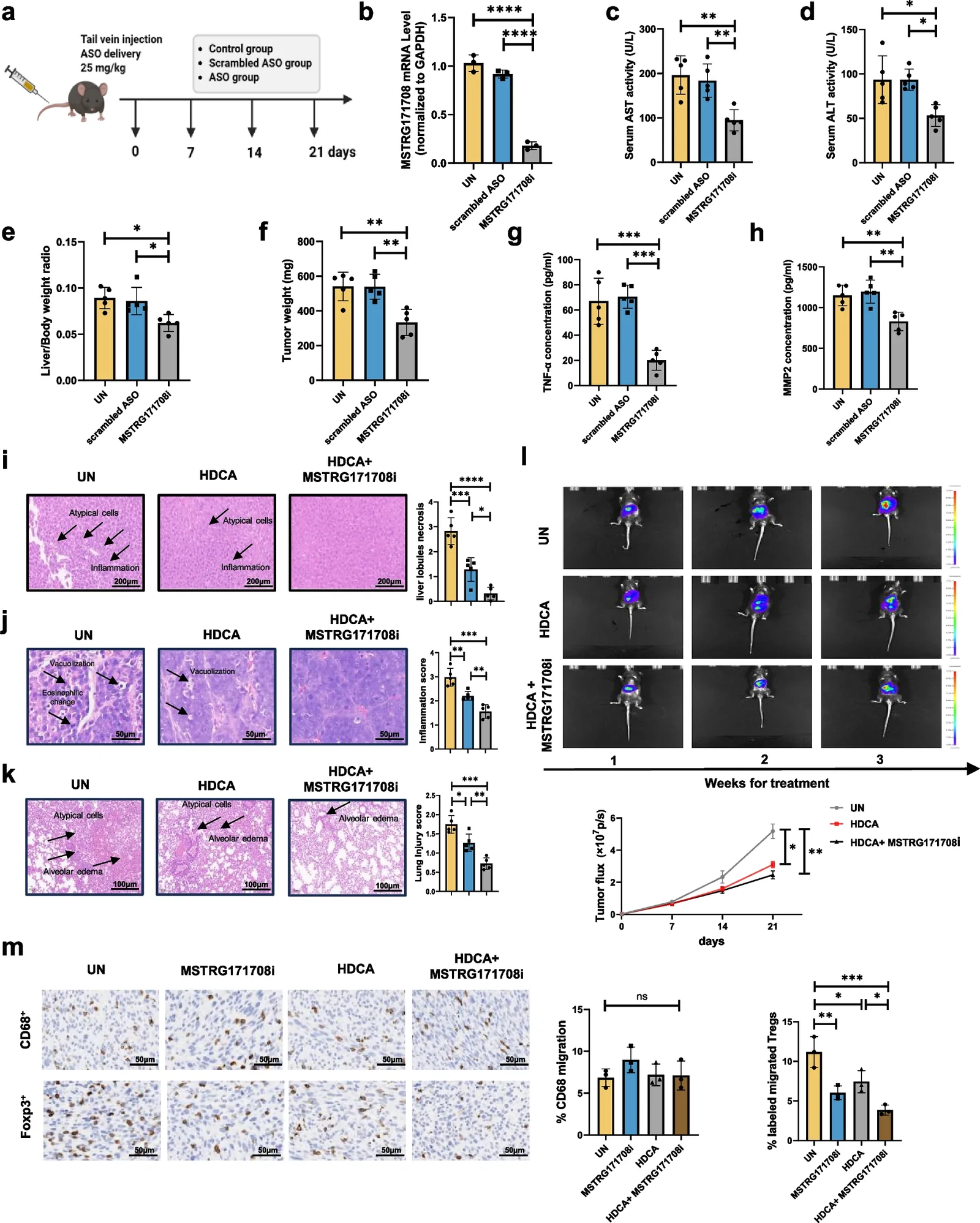
In vivo inhibition of lncRNA MSTRG171708 attenuates tumor progression and modulates immune microenvironment in HCC mouse models. **a** Schematic depiction of tail vein injection of antisense oligonucleotides (ASO) targeting MSTRG171708 in mice, administered at 25 mg/kg at specified time points over 21 days. Experimental groups include control, scrambled ASO, and ASO targeting MSTRG171708.** b** Quantification of MSTRG171708 RNA expression in liver tissue by RT-qPCR to evaluate lncRNA knockdown efficiency following ASO treatment. **c–d** Measurement of serum ALT and AST levels to evaluate liver function following lncRNA inhibition. **e–f** Quantification of Liver-to-body weight ratios and tumor weight in the indicated treatment groups.** g** ELISA quantification of serum TNF-α concentrations to assess pro-inflammatory cytokine production.** h** ELISA quantification of MMP2 concentration in serum with or without MSTRG171708i-treated mice. **i–j** Representative H&E images of liver tissues from mice subjected to different treatment conditions. Quantitative analysis of liver lobule necrosis and inflammatory infiltration. **k** Representative H&E images of lung tissues from different groups, showing atypical cells and alveolar edema (arrows). Lung injury scores are quantified on the right. **l** Longitudinal bioluminescence imaging tracks tumor burden and metastatic progression over the treatment period in different groups. **m** IHC analysis of CD68 + macropahges and FOXP3 + Treg in liver tumor tissues. Data are shown as mean ± s.d.. For panels b and m, *n* = 3 per group; for panels c–l, *n* = 5 per group. Statistical significance was determined by one-way ANOVA with post hoc tests or Student’s t-test, as appropriate. Ns, not significant. *p < 0.05, **p < 0.01, ***p < 0.001, ****p < 0.0001

To interrogate the functional relevance of MSTRG171708 in HCC progression and metastasis, we next established an orthotopic HCC mouse model and employed antisense oligonucleotides (ASOs) for targeted knockdown in vivo (Fig. 4a). Efficient lncRNA MSTRG171708 depletion was validated by RT-qPCR (Fig. 4b). Moreover, MSTRG171708 silencing led to improved liver function (Fig. 4c,d), decreased tumor burden as reflected by reduced liver-to-body weight and tumor weights (Fig. 4e,f), along with lower serum levels of pro-inflammatory factors TNF-α and the metastasis-associated marker MMP2 (Fig. 4g,h).

Histopathological assessments revealed that combined HDCA treatment and MSTRG171708 depletion profoundly alleviated hepatic injury, as demonstrated by significant reductions in lobular necrosis, inflammatory cell infiltration, and tissue architecture disruption (Fig. 4i,j). In the lungs, this combinatorial regimen substantially decreased lesion scores, signifying mitigation of metastatic outgrowth and distal organ injury (Fig. 4k). Longitudinal bioluminescence imaging further corroborated these findings, demonstrating robust and sustained suppression of tumor expansion and metastatic dissemination, underscoring the synergistic power of this therapeutic paradigm (Fig. 4l).

To further elucidate the effects of combination therapy on the tumor immune microenvironment, we performed immunohistochemical analysis of CD68⁺ macrophages and FOXP3⁺ Tregs. These markers were selected for their central roles in tumor-associated immunoregulation. Our results revealed that the abundance of CD68⁺ macrophages showed no significant changes between groups, while the proportion of infiltrating FOXP3⁺ Tregs was markedly reduced by HDCA and MSTRG171708 ASO treatments, with the most pronounced reduction observed in the combined treatment group, suggestting a selective decrease in immunosuppressive cell populations within the TME (Fig. 4m). Flow cytometric analyses further confirmed that dual treatment substantially reduced the proportion of immunosuppressiveFOXP3⁺CD25⁺ Tregs in both primary and metastatic tumor sites (Fig. S5d). In contrast, the proportions of CD4⁺ T cells (Fig. S5b), CD8⁺ T cells (Fig. S5c), neutrophils (Fig. S5a), macrophages (Fig. S5e), and dendritic cells (Fig. S5f) remained largely unchanged or showed only minor fluctuations across treatment groups. These findings indicate that combined targeting of MSTRG171708 and FXR signaling by HDCA robustly suppresses HCC progression and metastasis primarily by selectively depleting immunosuppressive Tregs within the TME, without broadly altering other major immune cell populations.

### LncRNA MSTRG171708 facilitates Treg recruitment and enhances immunosuppression in HCC

**Fig. 5:**
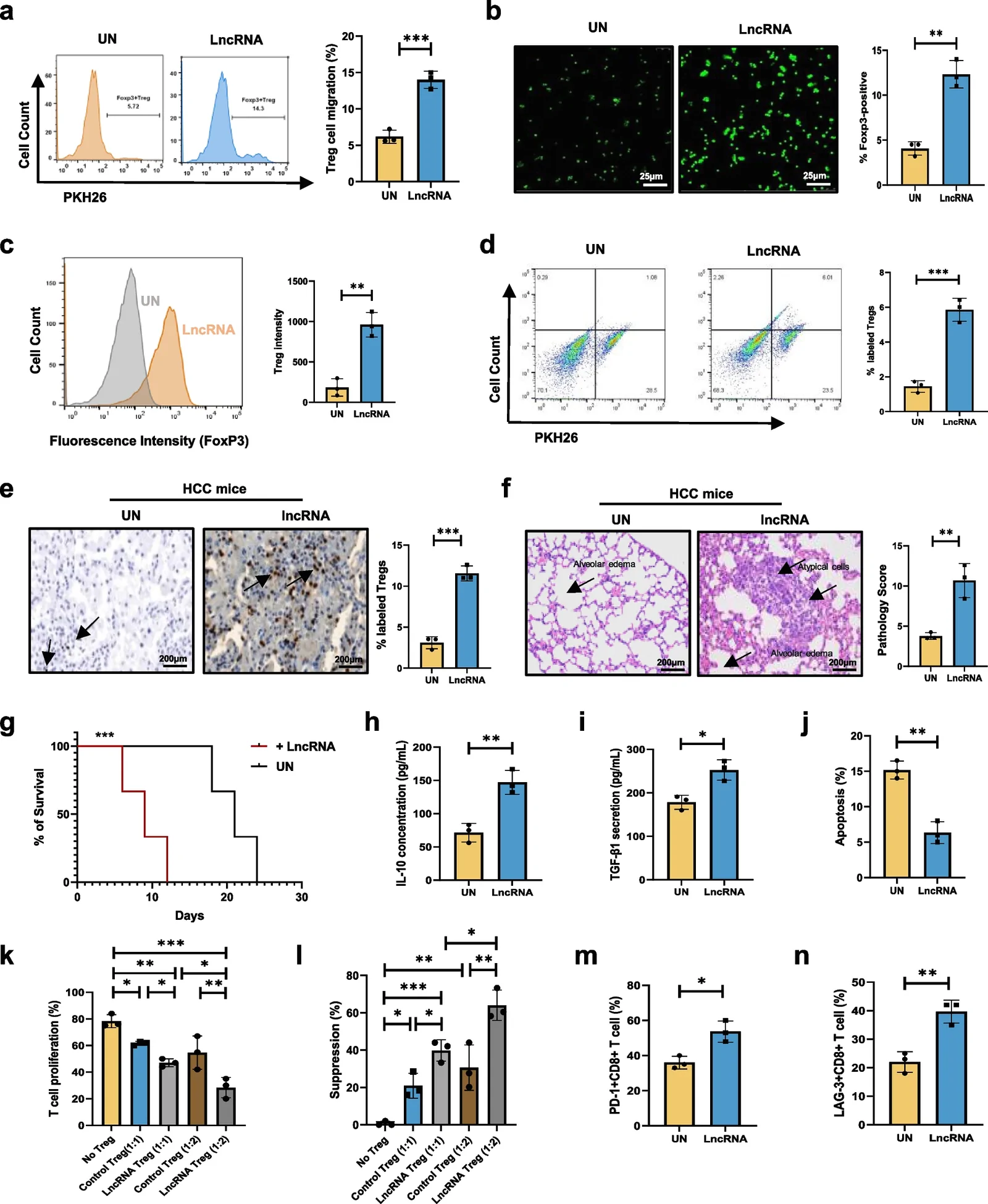
LncRNA promotes Treg migration, expansion, and immunosuppressive function in both in vitro and in vivo HCC models.A In vitro transwell migration assay was performed to assess Treg migration in untreated and LncRNA-treated group. B Representative fluorescence microscopy images and quantification showing that LncRNA significantly increases the proportion of Tregs compared to untreated controls. C Flow cytometric analysis of FoxP3 fluorescence intensity demonstrates increased Treg frequency in LncRNA-treated samples compared to untreated controls**. **D Flow cytometry was used to determine the proportion of PKH26-labeled Tregs following LncRNA MSTRG171708 treatment in tumor tissue.E IHC staining of HCC mouse tissues reveals increased infiltration of Tregs in LncRNA-treated mice.F Representative H&E staining and pathology scores of lung tissues from HCC mice, indicating exacerbated pathology in LncRNA-treated mice.G Kaplan–Meier survival analysis of HCC mice demonstrates reduced survival following LncRNA treatment. **h-i** Serum concentrations of IL-10 and TGF-β1 were determined by ELISA in LncRNA-treated and control mice.J Quantification of apoptosis percentage in hepatic tumor cells in untreated and LncRNA-treated groups.K T cell proliferation assay demonstrating decreased proliferation in co-culture with LncRNA-treated Tregs. L Suppression assay reveals enhanced suppressive capacity of LncRNA-treated Tregs. **m–n** Flow cytometric quantification of PD-1 + CD8 + and LAG-3 + CD8 + T cell populations, showing increased exhaustion markers in LncRNA-treated mice. All data are presented as mean ± s.d. (*n* = 3). Statistical significance was determined by one-way ANOVA with post hoc tests or Student’s t-test, as appropriate. Survival data were analyzed using the log-rank test. *p < 0.05, **p < 0.01, ***p < 0.001

To elucidate the role of EV-encapsulated lncRNA MSTRG171708 in modulating Treg recruitment and immunosuppression in HCC, we conducted a series of complementary in vitro and in vivo experiments. In Transwell migration assays, treatment with lncRNA MSTRG171708 markedly enhanced the migration of PKH26-labeled Tregs compared to untreated controls (Fig. 5a), a result corroborated by immunofluorescence staining, which showed increased accumulation of labeled Tregs upon lncRNA exposure (Fig. 5b). Consistently, flow cytometry revealed higher frequencies of FOXP3⁺ Tregs following MSTRG171708 treatment (Fig. 5c). In vivo administration of lncRNA MSTRG171708 led to a significant rise in both the abundance and percentage of labeled Tregs within hepatic tissue (Fig. 5d,e). Histopathological analysis demonstrated that mice treated with the lncRNA exhibited more pronounced tissue disruption and cellular infiltration in the lung, indicative of enhanced metastatic burden (Fig. 5f), while survival analysis indicated a significant reduction in overall survival following lncRNA exposure (Fig. 5g).

Further mechanistic interrogation revealed that lncRNA MSTRG171708 bolstered immunosuppressive signaling in HCC. ELISA assessment of serum cytokines demonstrated that lncRNA MSTRG171708 treatment markedly elevated the levels of the immunosuppressive cytokines IL-10 and TGF-β1 (Fig. 5h,i), and this was accompanied by a lower rate of tumor cell apoptosis (Fig. 5j), suggesting potentiated immune evasion. Functional assays further demonstrated that MSTRG171708-educated Tregs displayed augmented suppressive activity, as evidenced by decreased T cell proliferation and increased suppression rates in co-culture assays (Fig. 5k,l). Importantly, the lncRNA also increased the proportion of CD8⁺ T cells expressing the exhaustion markers PD-1 and LAG-3 (Fig. 5m,n), highlighting an impaired effector T cell response in the TME. Collectively, these findings demonstrate that MSTRG171708 facilitates Treg recruitment, expansion, and suppressive function, thereby promoting an immunosuppressive environment and effector T cell exhaustion in HCC.

### LncRNA MSTRG171708 promotes glycolysis-dependent Treg migration via PKM2/PHD3 interaction in theTME

**Fig. 6:**
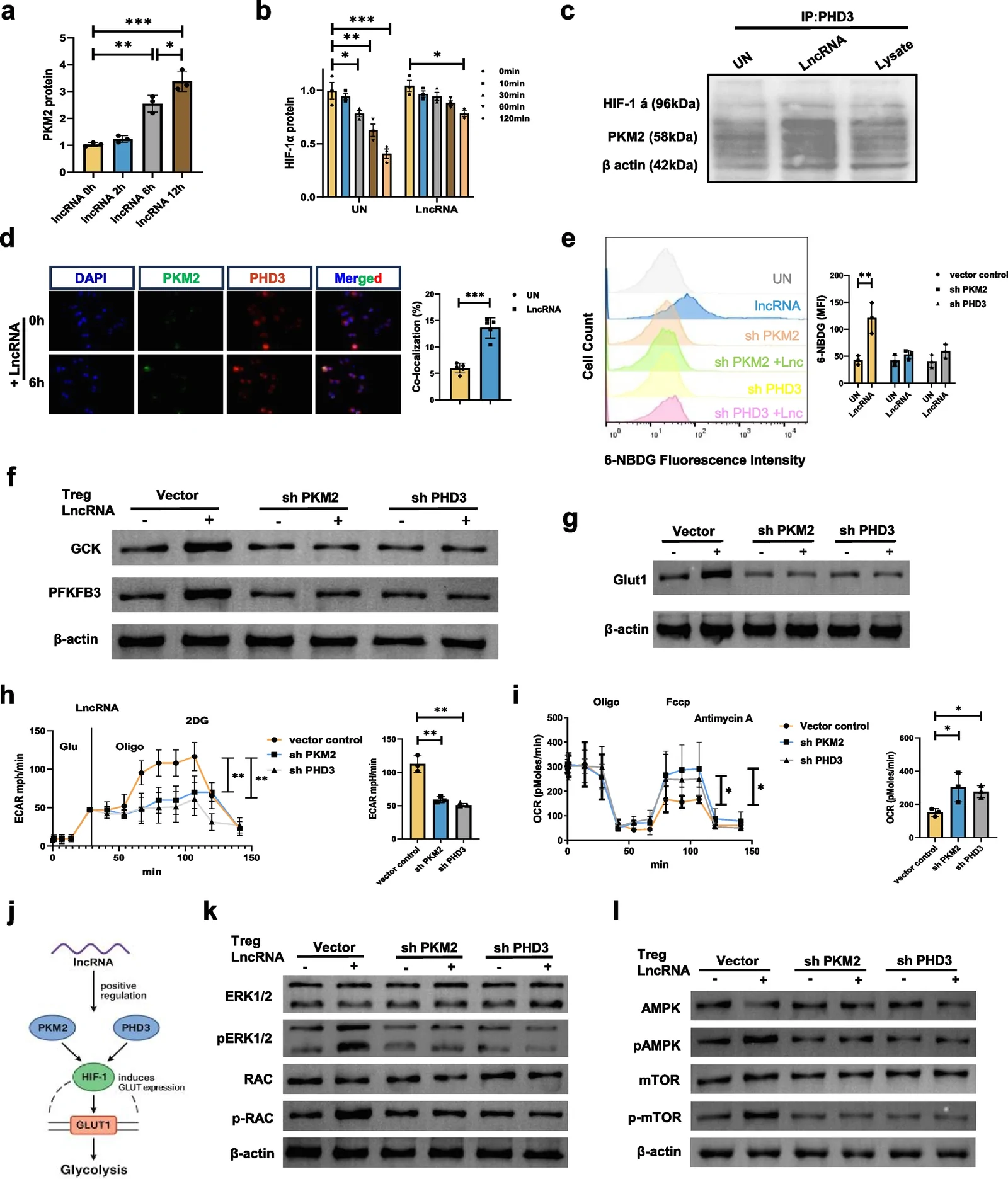
LncRNA promotes Treg cell glycolysis and migration via the PKM2–PHD3 axis. **a** Quantification of PKM2 protein levels in Tregs at indicated time points following lncRNA treatment. **b** Quantification of HIF-1α protein levels in Tregs over a time course in untreated and lncRNA-treated group. **c** Co-immunoprecipitation (co-IP) assay showing increased PKM2 and HIF-1α associated with PHD3 in lncRNA-treated Tregs compared to control. **d** Confocal analysis showing co-localization of PKM2 (green) and PHD3 (red) in Tregs at 0 h and 6 h after LncRNA stimulation. **e** 6-NBDG uptake assay demonstrating increased glucose uptake (MFI) in lncRNA-treated Tregs, which is reduced by shRNA-mediated PKM2 or PHD3 knockdown. **f** Immunoblot analysis of GCK and PFKFB3 in Tregs with or without shPKM2 or shPHD3 treatment. **g** Immunoblot and quantification of GLUT1 expression in Tregs; upregulation by lncRNA is reversed by shPKM2 or shPHD3. **h** ECAR demonstrating enhanced glycolysis in lncRNA- treated Tregs and abolished by shPKM2 or shPHD3 knockdown. **i** OCR showing a decrease in mitochondrial respiration after lncRNA treatment, partially rescued by shPKM2 or shPHD3. **j** Schematic summary of the proposed mechanism: LncRNA promotes the assembly of the PKM2/PHD3/HIF-1α complex, upregulates GLUT1, and driving glycolytic activity. **k** Immunoblotting showing increased ERK1/2 and RAC phosphorylation upon lncRNA treatment, attenuated by shPKM2 or shPHD3. **l** Immunoblot and quantification showing lncRNA-induced activation of AMPK and mTOR pathways, which is decreased by shPKM2 or shPHD3. Data are presented as mean ± s.d.; with the exception of d (*n* = 5), all experiments were performed in triplicate (*n* = 3). Statistical significance was assessed using an unpaired two-tailed t-test or one-way ANOVA with post hoc tests, as appropriate; *p < 0.05, **p < 0.01, ***p < 0.001

To unravel the mechanistic underpinnings of lncRNA MSTRG171708-mediated Treg migration and metabolic reprogramming in the TME, we focused on the interplay between PKM2 and PHD3, critical regulators of glycolytic activity [26, 27].Treatment of Tregs with lncRNA MSTRG171708 significantly elevated PKM2 protein levels and promoted stabilization of HIF-1α, a hallmark of enhanced glycolytic programming (Fig. 6a,b). Co-immunoprecipitation (co-IP) assays revealed that MSTRG171708 enhanced the association of both PKM2 and HIF-1α with PHD3 (Fig. 6c), indicating the assembly of a multi-protein complex central to metabolic regulation. Consistently, confocal imaging demonstrated increased co-localization of PKM2 and PHD3 following lncRNA stimulation (Fig. 6d), corroborating a role for MSTRG171708 in orchestrating protein complex formation. Strikingly, loss-of-function experiments using shRNAs targeting PKM2 or PHD3 abolished the lncRNA-driven upregulation of glucose uptake, Glut1 expression, and glycolytic enzymes GCK and PFKFB3 (Fig. 6e-g). Seahorse metabolic flux assays further substantiated that MSTRG171708-induced glycolytic acidification and suppressed oxygen consumption were strictly dependent on both PKM2 and PHD3 (Fig. 6h,i). In parallel, the metabolic alterations were accompanied by impaired migratory behavior following depletion of either factor (Fig. S6a,b). Mechanistically, lncRNA MSTRG171708 promotes the assembly of a PKM2/PHD3/HIF-1α complex, stabilizing HIF-1α and upregulating glycolytic gene expression, thereby enhancing Treg glycolytic programming and facilitating their migratory within the TME (Fig. 6j). This effect further supported by altered activation of the ERK/RAC migratory axis and the AMPK/mTOR metabolic pathway upon PKM2 or PHD3 depletion (Fig. 6k,l).

To further dissect the mechanisms underlying lncRNA MSTRG171708-mediated regulation of Treg metabolism and migration, we conducted both EV-mediated and cell-intrinsic assays. EVs from HDCA-treated tumor cells suppressed Treg glycolytic metabolism and migration, indicating that tumor-derived signals can impair Treg fitness through intercellular communication. Remarkably, reintroduction of lncRNA MSTRG171708 in tumor cells restored Treg metabolic activity and motility, highlighting the importance of EV cargo in shaping the Treg phenotype (Fig. S6c–f). Consistently, direct transfection of lncRNA in Tregs enhanced glycolysis and migration, whereas knockdown by ASO abrogated these functions (Fig. S6g–j). Collectively, these findings establish that lncRNA MSTRG171708 promotes glycolysis-dependent Treg migration via a PKM2/PHD3/HIF-1α axis and modulates the Treg phenotype both through tumor-derived EVs and cell-autonomous mechanisms, highlighting a central role for this lncRNA in coordinating immune cell metabolic and functional adaptation in HCC.

### MSTRG171708 modulates Treg transcriptional program and tumour glycolysis in HCC

**Fig. 7:**
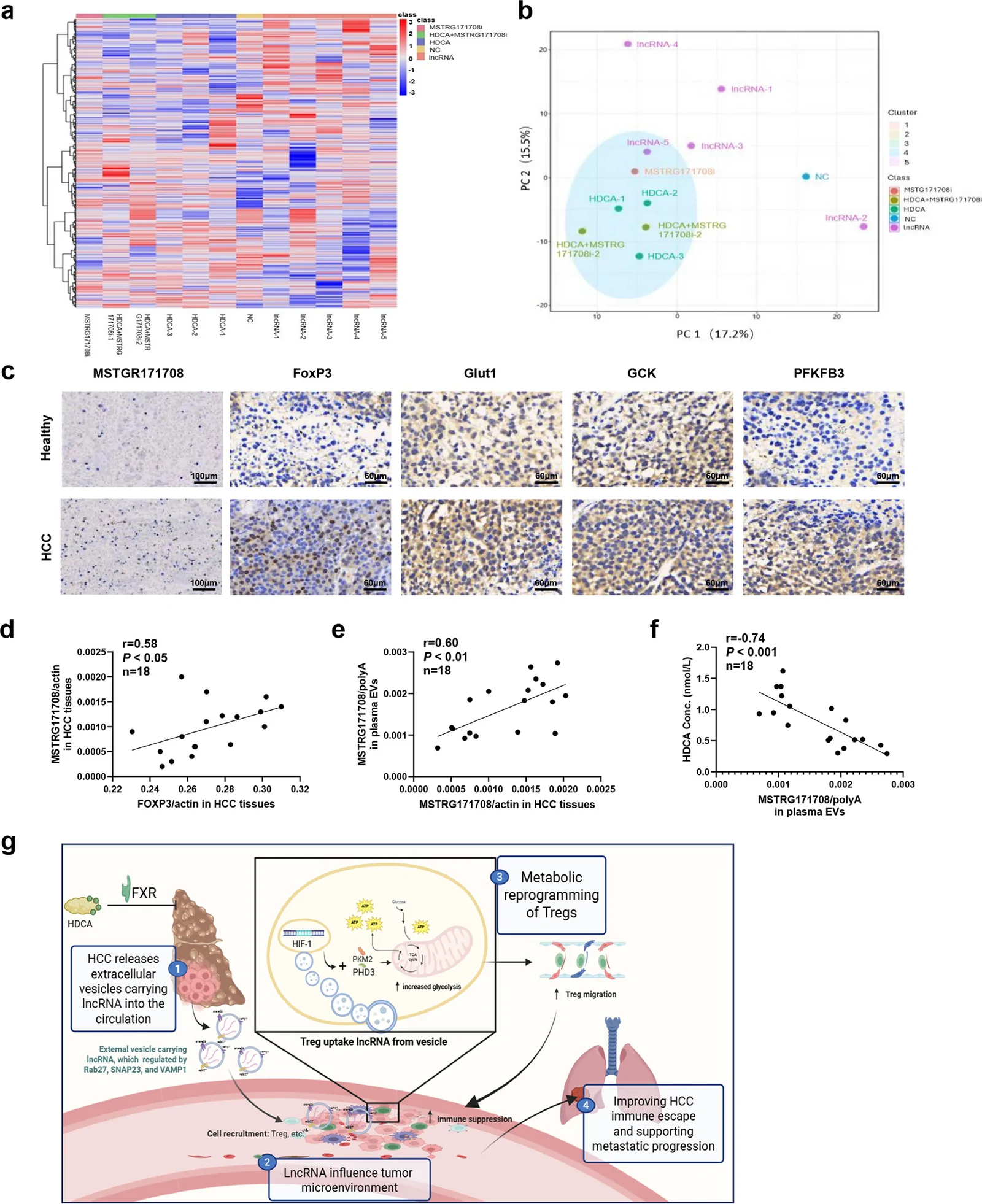
Differential expression and functional correlation of MSTRG171708 in HCC. **a** Hierarchical clustering heatmap of differentially expressed genes among the indicated groups (MSTRG171708i, HDCA + MSTRG171708i, HDCA, NC, lncRNA), showing distinct expression patterns. **b** Principal component analysis (PCA) of transcriptomic profiles demonstrates that the lncRNA group is clearly separated from other treatment groups along both PC1 and PC2.** c** Representative in situ hybridization (ISH) for MSTRG171708 and IHC for FoxP3, Glut1, GCK, and PFKFB3 in healthy and HCC tissues. **d** Correlation analysis between MSTRG171708 and FoxP3 expression in HCC tissues (*n* = 18, r = 0.58, *p* < 0.05). **e** MSTRG171708 expression in HCC tissues shows a strong positive correlation with its levels in plasma EVs (*n* = 18, r = 0.60, *p* < 0.01). **f** Negative correlation between MSTRG171708/polyA levels in plasma EVs and HDCA concentration in plasma (*n* = 18, r = –0.74, *p* < 0.001). **g** Schematic model illustrating the proposed mechanism by which HCC-derived EVs containing lncRNA MSTRG171708, released via Rab27/VAMP1/SNAP23-mediated exocytosis and inhibited by HDCA-FXR signaling, are taken up by Tregs in the TME. MSTRG171708 stabilizes HIF-1α, and promotes the assembly of the PKM2/PHD3/HIF-1α complex, driving glycolytic reprogramming and promoting immune suppression and metastasis

To comprehensively characterize the global transcriptional changes induced by lncRNA in Treg cells, we performed RNA sequencing analysis across Treg cells across HDCA treatment, MSTRG171708i, HDCA + MSTRG171708i, and lncRNA MSTRG171708 overexpression systems. Hierarchical clustering of differentially expressed genes revealed that the lncRNA-overexpressing group displayed a distinct transcriptomic profile compared to the HDCA-treated group, lncRNA-inhibition group, and NC controls (Fig. 7a). Principal component analysis (PCA) further demonstrated that lncRNA-overexpressing samples clustered separately from other groups, while HDCA-treated cells and those with lncRNA inhibition exhibited closer transcriptional similarity, underscoring the regulatory effect of this lncRNA on Treg programming (Fig. 7b).

To assess the clinical relevance of our findings, we examined MSTRG171708 expression and glycolytic marker profiles in primary HCC tissues using in situ hybridization (ISH) and immunohistochemistry. ISH analysis revealed a marked upregulation of MSTRG171708 in tumour specimens compared to healthy controls. Regions with elevated MSTRG171708 expression coincided with increased numbers of FoxP3⁺ Tregs and enhanced immunostaining for the glycolytic markers Glut1, GCK, and PFKFB3 (Fig. 7c). Further analysis demonstrated a positive correlation between MSTRG171708 expression and the abundance of FoxP3 + Tregs in HCC tissues, underscoring a potential link between MSTRG171708 and the immunosuppressive TME (Fig. 7d). In addition, levels of MSTRG171708 in plasma EVs exhibited a significant positive correlation with its expression in matched HCC tissue samples, suggesting that this lncRNA can be actively secreted into circulation as part of the tumor-immune cell crosstalk (Fig. 7e). Importantly, correlation analysis revealed a robust inverse relationship between HDCA concentration and MSTRG171708 expression in plasma, indicating that decreased HDCA levels are associated with increased secretion of MSTRG171708 by tumour cells (Fig. 7f). Together, these findings highlighted that MSTRG171708 may serve as a promising biomarker for disease progression and the immunometabolic state of the TME.

## Discussion

In this study, we delineate a novel regulatory axis in which HDCA ameliorates HCC progression and metastasis via inhibition of EV-mediated transfer of lncRNA MSTRG171708 and reducing the migration of Treg cells to tumor sites. Our data demonstrate that circulating HDCA is markedly reduced in HCC and correlates inversely with liver injury, tumor burden, and poor prognosis. Restoration of HDCA levels not only ameliorated liver pathology and suppressed tumor growth and metastasis but also remodeled the immune microenvironment and significantly improved survival in HCC-bearing mice, highlighting the translational potential of this BA in HCC management.

In agreement with emerging evidence that BAs function as critical metabolic and immune regulators in the liver TME [7], we identify HDCA as a pivotal modulator acting predominantly through the FXR. Previous research has mainly focused on FXR as a metabolic sensor or, in some instances, a tumour suppressor [28, 29]. Here, we show that HDCA exerts its anti-tumoral and anti-metastatic activities via FXR-dependent downregulation of pathological lncRNA and modulation of EV biogenesis, thereby inhibiting tumor growth and metastasis in HCC. Notely, the anti-tumor efficacy of HDCA being completely abolished in FXR-deficient models, demonstrating that HDCA’s protective action is strictly FXR-dependent. This finding highlights that both the skpecific ligand and the cellular context are critical in shaping FXR-mediated responses. In this way, our results help reconcile previous conflicting reports regarding FXR’s diverse roles in HCC, and further uncovers a mechanistic link between BA signaling and lncRNA/EV-mediated immunoregulation in the TME.

Our mechanistic study revealed that inhibits the transport of MVBs towards the plasma membrane by regulating lncRNA through Ras-related Rab-27 protein, which controls the docking process [30]. In addition, we identified that HDCA treatment disrupts the colocalization of Vamp1 with SNAP23, essential for SNARE complex formation and effective MVB fusion with the plasma membrane [31]. Concomitantly, HDCA treatment results in significantly decreased levels of EV-associated lncRNA MSTRG171708, indicating that HDCA-FXR signaling governs the selective export of regulatory lncRNAs, which may in turn restrict intercellular communication and attenuate the transmission of inflammatory cues between tumour cells and immune cells [32]. Importantly, EVs released from primary tumours can circulate to distant organs and contribute to the establishment of a premetastatic niche [33]. Our experimental results demonstrate that HDCA treatment inhibits the delivery of lncRNA MSTRG171708 mediated by tumour-derived EVs and suppresses liver tumour progression and metastasis, with an even more pronounced inhibitory effect observed when combined with lncRNA MSTRG171708 suppression.

Our study further demonstrate that lncRNA MSTRG171708 encapsulated in tumor-derived EVs reprogrammed the metabolism of Treg cells within the TME, thereby promoting their migration into hepatic tumor tissue and enhancing their immunosuppressive function. Mechanistically, MSTRG171708 enhances the protein abundance of PKM2, which facilitate its nuclear translocation and the assembly of a PKM2/PHD3/HIF-1α complex [34, 35]. This complex may acts as a coactivator to enhance HIF-1α-mediated transcription of glycolytic genes, thereby driving the metabolic adaptation of Treg cells in the TME [34, 36]. Our study significantly extends these findings by showing that tumor-derived exosomal MSTRG171708 intensifies this signaling axis in Treg cells. Notely, this modulation of protein activity and subcellular localization by MSTRG171708 exemplifies a classic mode of lncRNA action [37]. Furthermore, both PKM2 and PHD3 are themselves transcriptional targets of HIF-1α, implicating a positive feedback loop that further intensifies HIF-1α-driven metabolic adaptation [34, 38]. Supporting this, disruption of PKM2 or PHD3 abrogates the glycolytic and migratory enhancements conferred by MSTRG171708. These findings resonate with emerging literature on exosomal lncRNAs as key mediators of immune cell plasticity and cancer immune escape [39], and extend prior observations by demonstrating a direct mechanistic link between BA signaling, exosomal lncRNA packaging, and Treg metabolic adaptation in HCC.

Analysis of patient tissues reveals a robust inverse correlation between HDCA and MSTRG171708 levels, with EV-associated MSTRG171708 serving as a surrogate marker for Treg infiltration and glycolytic activation in the TME. These data suggest that combined assessment of HDCA and MSTRG171708 may provide a valuable biomarker pair for patient stratification and therapeutic monitoring. Furthermore, our data indicate that elevated MSTRG171708 promotes Treg recruitment, enhances immunosuppressive cytokine production, and contributes to T cell exhaustion—key features associated with an immunosuppressive TME and tumor progression. While our combinatorial intervention studies demonstrate that dual targeting of HDCA and MSTRG171708 results in synergistic anti-tumor and anti-metastatic effects. This supports the concept that simultaneous disruption of BA–exosome–lncRNA crosstalk may overcome immunotherapy resistance in HCC—a notion in line with recent work showing that targeting the BA–microbiota–immune axis can sensitize tumors to immune checkpoint blockade [40, 41].

Nonetheless, several limitations merit consideration. Our clinical validation cohort, while informative, was of moderate size and requires expansion across diverse populations to fully establish the prognostic and predictive value of HDCA and MSTRG171708. The influence of dietary, microbial, and host genetic factors on BA metabolism and EV cargo selection remains to be elucidated. Moreover, while our preclinical studies provide proof-of-concept for systemic HDCA administration and lncRNA inhibition, further investigation is needed to optimize delivery, dosing, and combinatorial strategies for clinical translation.

In conclusion, our work reveals a previously unappreciated pathway in which HDCA regulates HCC progression by modulating FXR-mediated repression of immunosuppressive lncRNAs in tumor-derived EVs, thereby reshaping the intratumoral immune microenvironment via Treg metabolic reprogramming **(**Fig. 7g**)**. These findings not only expand the understanding of BA signaling in cancer immunity but also highlight HDCA and the lncRNA MSTRG171708/EV axis as promising new targets for HCC immunotherapy. Pharmacological enhancement of HDCA or selective inhibition of lncRNA MSTRG171708 may provide a basis for innovative approaches to restrain Treg-mediated immune evasion and improve outcomes in HCC patients.

## Materials and methods

### Patients and clinic samples

Informed consent was obtained from all participants prior to enrollment. The study protocol was reviewed and approved by the ethics committee of the First Affiliated Hospital, Guangzhou University of Chinese Medicine (K-2025–083). All procedures involving human specimens were conducted in accordance with the Declaration of Helsinki and applicable ethical guidelines. Human HCC paraffin-embedded tissue and serum samples were anonymized and coded to protect patient confidentiality, following local ethical standards and regulatory requirements.

### Mice

All procedures involving animals were approved by the Institutional Animal Care and Use Committee of Hong Kong Baptist University (REC/23–24/0032) and conducted following relevant ethical guidelines. Male and female mice for HCC modeling and controls were maintained under specific pathogen-free (SPF) environments and utilized for experiments at 8–10 weeks of age. Group sizes were selected based on previous literature to ensure adequate statistical analysis. Mice were allocated to each experimental condition using a randomization protocol. Investigators responsible for data collection and interpretation were blinded to group allocation throughout the study.

### HCC model and treatment

Hepa1-6 cells stably expressing both GFP and luciferase (Hepa1-6-GFP/LUC) were used to establish orthotopic liver tumors in C57BL/6 J mice (*n* = 5 per group). Briefly, mice were anesthetized and the fur over the left liver lobe was removed. A small incision was made through the skin and abdominal musculature to expose the liver. Using a sterile 1 mL syringe, 2 × 10^6^ Hepa-1–6 L-luciferase cells in 25 µL of PBS were injected directly into the liver parenchyma. The incision was closed and mice were monitored until recovery. On Day 4 post-injection, mice began drug treatment. The treatment group received 100 mg/kg HDCA [6] in physiological saline by oral gavage twice weekly, while the control group received an equivalent volume of saline on the same schedule. For each imaging session, mice received 150 mg/kg D-luciferin via intraperitoneal injection and were anesthetized with isoflurane. After a 15-min incubation to allow for luciferin distribution, whole-body bioluminescent images were acquired using an IVIS Spectrum system (PerkinElmer). Body weight was recorded every three days, and survival was monitored daily throughout the intervention period.

### RNA sequencing and data processing

Total RNA was extracted from HCC with lung metastasis (HCC-LM), primary HCC (PHC), and healthy control (HC) tissues, including HDCA-treated groups, using TRIzol reagent (Invitrogen) according to manufacturer instructions. Ribosomal RNA was depleted with the Ribo-Zero rRNA Removal Kit (Illumina), and libraries were constructed using the TruSeq Stranded Total RNA Library Prep Kit (Illumina). Sequencing was performed on an Illumina NovaSeq 6000 with 150 bp paired-end reads. Raw sequencing reads were quality-checked using FastQC, and adapter sequences, low-quality bases (Q < 20), and reads shorter than 36 bp were removed with Trimmomatic. Clean reads were aligned to the human reference genome (GRCh38) using HISAT2, and transcript assembly and quantification were conducted with StringTie. Gene and lncRNA expression were normalized as fragments per kilobase per million mapped reads (FPKM). Differential expression analysis was performed with DESeq2, using |log2(fold change)|≥ 1 and adjusted p < 0.05 as significance cutoffs. The lncRNA MSTRG171708 was identified as a novel intergenic transcript (chr4:145,295,340–145,300,344, 5,005 bp, negative strand) with non-coding potential confirmed by CPC2, CPAT, and PhyloCSF. Conservation was assessed by PhastCons, and Pfam predicted a tetraspanin domain. Potential cis- and trans-acting target genes for MSTRG171708 were predicted based on expression correlation analysis (|Pearson r|≥ 0.7, adjusted p < 0.05) and genomic proximity; representative targets include Ccne1, Hk2, Tspan31, and Bcl2l1, suggesting its involvement in cell cycle regulation, glycolysis, apoptotic processes, and metastasis. Subcellular localization prediction using lncLocator2 and iLoc-lncRNA suggested predominant cytoplasmic distribution.

### Isolation of Treg cells

Treg cells were isolated from the spleens and lymph nodes of mice using magnetic-activated cell sorting (MACS). Briefly, single-cell suspensions were prepared by mechanical dissociation and filtration through a 70-μm cell strainer. CD4 + T cells were first enriched using anti-CD4 microbeads (Miltenyi Biotec), according to the manufacturer’s instructions. The CD4 + cell fraction was then labeled with anti-CD25 microbeads, and CD4 + CD25 + cells were further purified by positive selection using a MACS separator. The purity of Treg cells was confirmed by flow cytometric analysis of CD4, CD25, and Foxp3 expression, and was consistently ≥ 90%.

### Isolation and characterization of EVs

EVs were isolated from mouse plasma, tumor cell culture media, and tumor tissues using adapted standard protocols. Culture supernatants were collected after 24–48 h of serum-free incubation, cleared by sequential centrifugation (3,000 rpm for 10 min; 10,000 × g for 30 min, 4 °C), filtered (0.22 μm), and subjected to ultracentrifugation (100,000 × g for 90 min) to pellet EVs. Plasma was obtained by centrifuging anticoagulated blood at 2,000 × g for 15 min at 4 °C, treated with thrombin, centrifuged at 10,000 × g for 30 min, filtered, and EVs were purified using ExoQuick Ultra kit (System Biosciences). Tumor tissues were minced, enzymatically digested (collagenase D, DNase I, 30 min, 37 °C), filtered (70 μm), and the supernatant was cleared by stepwise centrifugation and filtration prior to ultracentrifugation (100,000 × g for 90 min). All EVs underwent additional purification by OptiPrep density gradient (5%–40% iodixanol, 100,000 × g, 18 h, 4 °C). EV morphology was analyzed by TEM and EV identity was confirmed by western blot analysis for CD9 (555,370, BD Biosciences), CD63 (sc-5275, Santa Cruz), CD81 (MAB4615, R&D), and TSG101(125,011, Abcam). Calnexin (ab22595, Abcam) and GM130 (11,308–1-AP, Proteintech) served as negative controls.

### Co-incubation of EVs with Hepa1-6 and Tregs

EVs were isolated from the conditioned medium of Hepa1-6 cells. EVs were incubated with 2 μl PKH26 dye (Sigma) for 5 min at room temperature in the dark. The labeled EVs were washed via ultracentrifugation at 120,000 × g for 2 h and subsequently purified using exosome spin columns (MW 3000, Invitrogen) to eliminate unbound dye. The PKH26-labeled EVs were resuspended in sterile-filtered PBS and added to Treg cells at a concentration of 10 μg/ml in CSC complete medium in 12-well plates, followed by incubation for 24 h at 37 °C. Cellular nuclei were counterstained with DAPI (blue). The uptake of EVs by Treg cells was visualized and analyzed using fluorescence microscopy (Essen IncuCyte ZOOM, Essen BioScience), with both phase-contrast and fluorescence images acquired every 3 h over the 24-h period.

### Immunohistochemistry

Tissue samples embedded in paraffin were sectioned at a 4 μm thickness, then cleared of paraffin using xylene and gradually rehydrated via descending concentrations of ethanol. Heat-induced antigen unmasking was carried out in 0.01 M citrate buffer (pH 6.0) using a high-pressure device. After cooling, tissue sections were incubated with 3% hydrogen peroxide to suppress endogenous peroxidase activity, and subsequently blocked with 5% goat serum for half an hour to minimize nonspecific binding. Primary antibodies targeting Rab27 (1:100, Abcam ab184996), Vamp1(1:200; Abcam, ab215180), SNAP23 (1:100; Abcam, ab180116), FoxP3 (1:100, Abcam ab200034), CD68 (1:100, Abcam ab31630), Anti-Ki67 antibody (1:200, Abcam, ab15580,), PCNA(1:200, CST #13,110), GLUT1(1:100, Abcam, ab115730), GCK(1:100, Abcam, ab37796), PFKFB3 (1:200, Abcam, ab181861) were applied and allowed to react overnight at 4 °C. After rinsing with PBS, the slides were exposed to HRP-labeled secondary antibodies for one hour at ambient temperature. Immunoreactivity was visualized via a diaminobenzidine (DAB) substrate and nuclei were then stained with hematoxylin prior to a final wash. For quantification, five high-power fields were chosen at random from each section for evaluation. The H-score was calculated as follows: H-score = [1 × (% of cells with weak staining)] + [2 × (% of cells with moderate staining)] + [3 × (% of cells with strong staining)], yielding a range of 0–300.

### Transfection and transduction

For in vivo knockdown, 2'-O-methyl phosphorothioate antisense oligonucleotides (ASOs; 18–22 nt) targeting exonic sequences of MSTRG171708 were designed using IDT OligoAnalyzer and verified for specificity against the mouse genome with BLAST. Scrambled ASOs lacking homology served as negative controls. All ASOs were synthesized by BGI (Shenzhen, China), dissolved in sterile PBS, and administered to Hepa1-6-GFP/LUC mice via tail vein injection (25 mg/kg, once weekly for three weeks). Mice were sacrificed and tissues harvested on day 21 post-initial injection for downstream analyses. ASO efficacy and specificity were validated by BGI prior to in vivo use. For overexpression, the full-length MSTRG171708 cDNA was cloned into a CMV-driven lentiviral vector. Lentivirus was produced by co-transfecting HEK293T cells with the expression construct plus packaging plasmids psPAX2 and pMD2.G. Virus-containing supernatants were collected, filtered, and used to infect Hepa1-6 cells with 8 μg/mL polybrene. For in vivo studies, concentrated lentiviral particles were delivered to mice via tail vein injection. For gene silencing in Treg cells, 5 × 10^5^ cells per well were spinfected with shRNA-encoding lentivirus (targeting PKM2 or PDH3; MOI = 100) and 5 μg/mL polybrene (800 × g, 60 min, room temperature). Media was changed after 24 h, and cells were cultured for an additional 48–72 h. Knockdown and transduction efficiency were assessed by RT-qPCR.

### Confocal microscopy

A total of 1 × 10^5^ cells were transferred into 24-well plates containing glass coverslips (VWR International) pretreated with 50 µg/ml poly-D-lysine (Gibco). Cells were cultivated overnight at 37 °C in 5% CO₂ to allow formation of a monolayer. Subsequently, cells were fixed with 4% paraformaldehyde (Sigma-Aldrich) for 20 min at 4 °C. Following fixation, samples underwent PBS washes and stained with SNAP23 (1:100; CST, 10,997), Vamp1 (1:100; Abcam, ab619320), PKM2 (1:100; CST, 4053), PHD3 (1:100; CST, 3436) antibodies for 4 h at room temperature, washed, and incubated with species-specific Alexa Fluor-labeled secondary antibodies for 1 h. After washing, nuclei were counterstained with DAPI (10 min), followed by mounting with VECTASHIELD mounting medium (Vector Laboratories, H-1800). Fluorescent images were acquired using a Zeiss LSM800 confocal microscope.

### Measurement of extracellular acidification rates (ECAR) and oxygen consumption rates (OCR)

ECAR and OCR of Treg cells were measured using the Seahorse XF analyzer (Seahorse Biosciences). Treg cells were cultured in serum-free, unbuffered XF assay medium (Cat#102,365–100) for 1 h and seeded at 6 × 10^5^ cells/well in XF24 plates. Metabolic modulation was assessed by sequential addition of oligomycin (1 μM), FCCP (1 μM), antimycin A (1 μM), rotenone (1 μM), D-glucose (10 mM), and 2-deoxy-D-glucose (2DG, 50 mM; all from Seahorse Biosciences). Assays were performed with 2 min mixing, 2 min waiting, and 4–5 min measurement intervals. Data were analyzed using Wave v2.4.1 software.

### Flow cytometry

Cells were resuspended in FACS buffer containing PBS, bovine serum albumin, and sodium azide. After incubation with fluorochrome-labeled antibodies or corresponding isotype controls, samples were processed according to the manufacturer's protocol. Cell fixation was performed using a paraformaldehyde-based solution supplemented with fetal calf serum. Flow cytometric analysis was conducted on a FACS Aria instrument (Becton Dickinson), and data acquisition was subsequently processed using FlowJo version 10.8 (Tree Star).

### Statistics and reproducibility

Statistical analyses were performed using SPSS v13.0 or GraphPad Prism 10.0. Data are presented as mean ± s.d.. Two-group comparisons used the unpaired two-tailed Student’s t-test. Multiple group comparisons used one-way ANOVA with Tukey’s post hoc test. Pearson’s correlation coefficient was used for correlation analysis. Survival analysis was performed using the Kaplan–Meier method and compared using log-rank tests. All experiments were repeated three or five times.

## Supplementary Information

Supplementary Material 1.

## Data Availability

The code and data for this study are available at https://github.com/kcpcheung/MSTRG171708-. Additional data are available to qualified researchers upon reasonable request by contacting kcpcheung@hkbu.edu.hk. Access requires a formal proposal and approval from the institutional ethics and data management committees.

## Abbreviations

HCC: Hepatocellular carcinoma
HDCA: Hyodeoxycholic acid
EV: Extracellular vesicle
lncRNA: Long non-coding RNA
FXR: Farnesoid X receptor
TME: Tumor microenvironment
Treg: Regulatory T cell
ASO: Antisense oligonucleotide
ECAR: Extracellular acidification rate
OCR: Oxygen consumption rate
ANG1: Angiopoietin-1
VEGF: Vascular endothelial growth factor
HIF-1α: Hypoxia-inducible factor 1 alpha
PKM2: Pyruvate kinase M2
PHD3: Prolyl hydroxylase domain-containing protein 3
Rab27: Ras-related protein Rab-27
