# Supplementary material for: HDCA suppresses exosomal lncRNA MSTRG171708 release to inhibit Treg-mediated immunometabolism in hepatocellular carcinoma
Source: Mol Biomed. 2026 Sep 11;7:168. doi: 10.1186/s43556-026-00550-0 (PMC13569752; doi:10.1186/s43556-026-00550-0)
Supplement: Supplementary file 1 [file 43556_2026_550_MOESM1_ESM.docx]

**Supplementary information**

**Title:** HDCA Suppresses Exosomal lncRNA MSTRG171708 Release to Inhibit Treg-Mediated immunometabolism in Hepatocellular Carcinoma

Feng Yang^1#^, Wenqiong Huang^2#^, Chongkai Fang^3^, Chong Zhong^4^, Chaoyuan Huang^5^, Meijun Liu^2^, Zongzhen Meng^2^, Xiaoli Chen^2^, Claudio Mauro^6*^, Aiping Lyu^2*^, Kenneth CP Cheung^2*$^

**Author information**

^1^ The First Affiliated Hospital, Zhejiang University School of Medicine, Hangzhou, Zhejiang Province, China

^2^ Phenome Research Center, Hong Kong Baptist University, Hong Kong, China

^3^ Science and Technology Innovation Center, Guangzhou University of Chinese Medicine, Guangzhou, China.

^4^ Department of Biliary-Pancreatic Surgery, The First Affiliated Hospital of Guangzhou University of Chinese Medicine, Guangzhou, China.

^5^ Department of Gastroenterology, Guangdong provincial hospital of Chinese medicine, Guangzhou, China

^6^ Department of Inflammation and Ageing, College of Medicine and Health, Queen Elizabeth Hospital, Mindelsohn Way, B15 2WB, University of Birmingham

^#^ Equal contributions

^*^ Corresponding authors: C Mauro c.mauro@bham.ac.uk, A Lyu aipinglu@hkbu.edu.hk, Kenneth CP Cheung kcpcheung@hkbu.edu.hk

^$^ Lead contact: kcpcheung@hkbu.edu.hk

**Fig. S1** Differential BA profiles and the effect of HDCA in HCC models. **a–b** Serum BAs composition in healthy controls (n=12) and HCC patients (n=20). **c** Ranking of Mean Decrease Accuracy of BAs via Random Forest Model. **d** Heatmap showing correlations between serum BAs and clinical parameters (ALT, AST, AFP, TG, H-Score) in HCC patients. **e** Hepatic HDCA concentration in mice from vehicle, HCC, and HDCA treatment groups. **f** Quantification of VEGF-A expression in mouse liver tissue by qRT-PCR. **g** Relative expression of PCNA in mouse livers assessed by qRT-PCR. **h** Percentage of proliferating hepatocytes measured by Ki-67 IHC staining. **i** TUNEL assay quantification of apoptotic cells in liver tissues. Data are presented as mean ± s.d.. n = 3 for (f-i); n = 5 for (e). Statistical significance were determined by one-way ANOVA with Tukey’s post hoc test. **p* < 0.05, ***p* < 0.01, ****p* < 0.001.

**Fig. S2** Impact of FXR regulation on tumorigenesis and metastasis. **a** Quantitative PCR analysis of FXR and TGR5 mRNA expression in liver tissues from mice treated with HDCA or untreated controls. **b-c** Relative mRNA levels of SHP and BSEP in liver tissue from HDCA-treated and untreated mice. **d** Relative mRNA levels of DIO2 in liver tissue from HDCA-treated and untreated mice. **e** Representative H&E-stained liver sections from vector control and FXR KO mice treated with or without HDCA, with quantification of pathology scores shown on the right. **f** Quantification of lung metastatic nodules in vector control and FXR KO mice, with or without HDCA treatment. **g** Representative images and quantification percentage of Arginase-1 positive cells in lung tissue from vector control and FXR KO mice, with or without HDCA treatment. **h** Macroscopic images of liver tumors and quantification of tumor volume and weight in vector control and FXR OE mice, with or without HDCA treatment. **i** Representative IHC staining of liver tissues with PCNA in vector control and FXR OE mice, with quantification of H-score and Lesion area. **j** Bioluminescence images and quantification of Hepa-Luc signals in vector control and FXR OE mice after HDCA with or without HDCA treatment. Data are presented as mean ± s.d., with the exception of f and h (n = 5), all experiments (a–j) were performed in triplicate (n = 3). Statistical significance was assessed by Student’s t-test or one-way ANOVA. **p* < 0.05, ***p* < 0.01, ns: not significant.

**Fig. S3** Differential expression and functional analysis of lncRNAs and mRNAs in mouse HCC models. **a-b** Hierarchical clustering heatmaps were generated from RNA sequencing data to visualize the expression profiles of (a) 5,077 novel lncRNAs and (b) 23,478 mRNAs in mouse serum EVs. HCC-LM refers to hepatocellular carcinoma with lung metastasis; PHC denotes primary hepatic carcinoma; and HC represents healthy controls. **c-e** Comparative analysis was conducted between two groups using volcano plots to identify differentially expressed genes, providing a visual representation of the expression variation. **f-h** Bar plots showing the number of differentially expressed (f) mRNAs, (g) lncRNAs, and (h) total transcripts. **i-k** A Venn diagram was used to analyze the intersection of differentially expressed genes between the two groups, revealing 20 genes with consistent differential expression across all comparisons, including 4 novel lncRNAs and 16 mRNAs. **l** Cellular component enrichment analysis of differentially expressed lncRNAs reveals significant enrichment in vesicle-related structures, with bubble size indicating gene counts and color reflecting statistical significance. **m** RNA FISH analysis demonstrates that MSTRG171708 (red) is predominantly localized in the cytoplasm of HCC cells. Nuclei are counterstained with DAPI (blue), and the cytoskeleton is stained with FITC-conjugated anti-actin antibodies (green). Scale bar, 10 μm.

**Fig. S4** FXR regulates EV secretion and vesicular transport pathway in tumor cells. **a** Schematic illustration of EV biogenesis and release pathway. **b** Representative TEM images of EVs isolated from HCC cells treated with or without HDCA, and quantification of EV particle number per field (right). Scale bar, 100 nm. **c** Relative expression of LncRNA MSTRG171708 in HCC cells with or without HDCA stimulation and TGR5 KO. **d** Relative expression of CD63 in HCC cells treated with TGR5 vector, TGR5 KO, and TGR5 OE, showing no significant differences. **e** Expression levels of MSTRG171708 in HCC-derived EVs following treatment with control IgG or anti-CD81 neutralizing antibody. **f** Relative expression of MSTRG171708 in cell lysate, culture supernatant, isolated EVs, and EVs treated with RNase, highlighting MSTRG171708 is enclosed within EVs rather than associated with their external surface. **g** Relative MSTRG171708 expression in EVs after treatment with RNase, Triton, or Triton combined with RNase reveals that lncRNA is sensitive to RNase only after EV membrane disruption by Triton. **h** Immunoblot analysis of calnexin and GM130 in whole cell lysate (WCL), EVs isolated from the plasma of HCC mice (EVs-HCC), and EVs isolated from the plasma of healthy mice (EVs-Normal). Quantitative analysis is shown below. **i** Immunoblot analysis of Rab27, SNAP23, and Vamp1 in tumor cells with vector control or FXR KO under untreated or HDCA conditions; quantification is shown on the right. **j** Immunofluorescence staining of DAPI (blue), Vamp1 (green) and SNAP23 (red) in tumor cells with vector control or FXR KO under UN or HDCA. Scale bar, 5 μm. Quantification of co-localisation is shown on the right (n = 500 cells per group). **k-l** Knockdown of Rab27 and SNAP23 significantly reduced MSTRG171708 expression compared to control and shGFP-treated cells. **m** Uptake of secreted lncRNA by Tregs measured by qRT-PCR after co-incubation with EVs. Data are presented as mean ± s.d. (n=3). Statistical significance was determined by one-way ANOVA with Tukey’s post hoc test or two-tailed unpaired t-test as appropriate. **p* < 0.05, ***p* < 0.01, ****p* < 0.001, *****p* < 0.0001; ns, not significant.

**Fig. S5** Flow cytometric characterization of tumor-infiltrating immune cell populations in liver primary and metastatic tumors following HDCA and lncRNA MSTRG171708 inhibition. **a** Flow cytometry analysis of tumour-infiltrating CD11B⁺Ly6G⁺ (neutrophils) in liver primary tumor tissues and metastatic tumor tissues to assess the impact of HDCA and MSTRG171708 inhibition on the accumulation of these immunosuppressive myeloid cells. **b** CD4+ T cell proportions in liver tumor tissues were measured to evaluate changes in helper T cell populations following treatment. **c** CD8+ T cell proportions were analyzed to determine potential cytotoxic T cell modulation. **d** Tregs were assessed in primary and metastatic tumors under different treatment conditions, including HDCA alone and in combination with lncRNA MSTRG171708 inhibition. **e** The proportions of macrophage and monocytic MDSCs (Mo-MDSCs) were measured to examine the influence of HDCA and lncRNA MSTRG171708 inhibition on myeloid cell subsets. **f** Dendritic cells (DCs) were quantified to assess antigen-presenting cell dynamics under the various treatment conditions. Data are presented as representative flow cytometry plots and quantitative group comparisons (right panels). n = 3 per group. Statistical significance was determined by one-way ANOVA with post hoc test. **p* < 0.05, ***p* < 0.01.

**Fig. S6** LncRNA facilitates Treg glycolysis and migration. **a** Fluorescence intensity of migrated Tregs is increased by lncRNA, but reduced with shPKM2 or shPHD3 knockdown. **b** Migrated cell count (per field) demonstrates enhanced Treg migration with lncRNA, attenuated by PKM2 or PHD3 silencing. **c-f** Treg cells were incubated with EVs from untreated tumor cells (control-EV), HDCA-treated tumor cells (HDCA-EV), HDCA-treated tumor cells transfected with synthetic lncRNA MSTRG171708 (HDCA-EV + lncRNA MSTRG171708), or HDCA-treated tumor cells with control RNA (HDCA-EV + control RNA). Shown are (c) ECAR (mpH/min), (d) glucose uptake (mean fluorescence intensity, MFI), (e) lactate production, and (f) Treg migration (number of migrated cells per field). HDCA-EV impaired glycolytic activity and migration of Tregs, while supplementation with lncRNA MSTRG171708 significantly restored these functions. **g-j** ECAR (g), glucose uptake (h), lactate production (i), and number of migrated Treg cells (j) were measured in control Tregs, Tregs transfected with lncRNA MSTRG171708, scrambled ASO, or lncRNA MSTRG171708-targeting ASO. Transfection of lncRNA MSTRG171708 enhances glycolysis, glucose uptake, lactate production, and Treg migration, whereas inhibition of lncRNA MSTRG171708 suppresses these effects. Bar graphs represent mean ± s.d.; n=3. Statistical significance was determined by one-way ANOVA with post hoc test; **p* < 0.05, ***p* < 0.01, ****p* < 0.001.
